# Immune checkpoint molecules are regulated by transforming growth factor (TGF)-*β*1-induced epithelial-to-mesenchymal transition in hepatocellular carcinoma

**DOI:** 10.7150/ijms.54239

**Published:** 2021-04-22

**Authors:** Ritu Shrestha, Kim R. Bridle, Darrell H. G. Crawford, Aparna Jayachandran

**Affiliations:** 1 Faculty of Medicine, The University of Queensland, Brisbane, Queensland, Australia.; 2 Gallipoli Medical Research Institute, Greenslopes Private Hospital, Brisbane, Queensland, Australia.; 3 Fiona Elsey Cancer Research Institute, Ballarat, Victoria, Australia.

**Keywords:** immune checkpoint molecules, epithelial-to-mesenchymal transition, transforming growth factor-β1, hepatocellular carcinoma

## Abstract

Hepatocellular carcinoma (HCC) is the most common type of primary liver cancer with a high mortality rate. Epithelial-to-mesenchymal transition (EMT) confers cancer cells with immune evasive ability by modulating the expression of immune checkpoints in many cancers. Thus, the aim of our study is to examine the interplay between EMT and immune checkpoint molecules in HCC. A reversible EMT model was utilised with transforming growth factor (TGF)-*β*1 as an EMT inducer for HCC cell lines Hep3B and PLC/PRF/5. HCC cells were treated with TGF-*β*1 for 72 h and the EMT status and immune checkpoint expression were examined. In addition, the migratory ability of HCC cells were examined using wound healing and transwell migration assays in the reversible EMT model. siRNA-mediated knockdown of immune checkpoint molecule, B7-H3, was further utilised to validate the association between TGF-*β*1-mediated EMT and immune checkpoint expression in HCC. In addition, a web-based platform, SurvExpress, was utilised to evaluate the association between expression of TGF-*β*1 in combination with immune checkpoint molecules and overall survival in HCC patients. We observed induction of EMT upon treatment of HCC cells with TGF-*β*1 revealed by reduced expression of epithelial markers along with increased expression of mesenchymal markers. Withdrawal of TGF-*β*1 reversed the process of EMT with elevated expression of epithelial markers and reduced expression of mesenchymal markers. TGF-*β*1 treatment elevated the migratory potential of HCC cells which was reversed following reversal assay. Notably, during TGF-*β*1-induced EMT, there was upregulation of immune checkpoint molecules PD-L1 and B7-H3. However, the reversal of EMT decreased the expression of PD-L1 and B7-H3. In addition, TGF-*β*1 driven EMT was reversed following knockdown of B7-H3 in both HCC cells further validating the interplay between TGF-*β*1-mediated EMT and immune checkpoint expression in HCC. Furthermore, the coordinate expression of TGF-*β*1 with PD-L1 (*p=0.01487*) and B7-H3 (*p=0.009687*) was correlated with poor overall survival in 422 HCC patients. Our study has demonstrated a close association between TGF-*β*1-mediated EMT and regulation of immune checkpoints in HCC.

## Introduction

Hepatocellular carcinoma (HCC) constitutes the majority of primary liver cancer, which is one of the leading causes of cancer-related deaths and the sixth most common cancer based on incidence 1, 2. HCC is a rapidly growing second most lethal tumour with rate of mortality almost similar to the rate of incidence 1-4. HCC is frequently diagnosed in cirrhotic patients with higher risk in patients with underlying liver disease resulting mainly from hepatitis B or C virus infection 2.

Sorafenib and Lenvatinib are the Food and Drug Administration (FDA) approved first-line drugs for the treatment of advanced HCC. Regorafenib, Ramucirab and Cabozantinib are the second-line therapies for HCC patients previously treated with Sorafenib. In addition, immunotherapeutic approaches have been explored in recent years as an alternative second-line treatment modality in HCC. Cancer immunotherapy based on immune checkpoint inhibition has transformed oncology treatment in several cancers including HCC 5-7. Immune checkpoint inhibitors (ICIs) blocking programmed cell death protein-1 (PD-1) such as Nivolumab and Pembrolizumab have been approved for treatment of HCC. Recently, the FDA has approved the combination of Nivolumab with another checkpoint inhibitor, Ipilimumab that targets cytotoxic T-lymphocyte-associated protein 4 (CTLA-4), for treatment of patients with advanced HCC previously treated with Sorafenib 8. In addition, the FDA has also approved the combination of Atezolizumab, ICI against programmed cell death protein ligand -1 (PD-L1), and Bevacizumab as first-line treatment for HCC patients based on the response rates in the IMbrave150 trial 9. Studies have demonstrated that immune checkpoint molecules are often overexpressed in HCC patients with poor prognosis 10, 11. However, the precise mechanism associated with regulation of immune checkpoint molecules in HCC is still unknown.

The process of epithelial-to-mesenchymal transition (EMT) is a key step in cancer metastasis and invasion that allows phenotypic conversion of cells to gain migratory and invasive features 12-14. EMT is a major contributor to disease progression and drug resistance in HCC 13, 15. In addition, the reverse process of mesenchymal-to-epithelial transition (MET) allows tumour recurrence post migration and invasion that is facilitated through the process of EMT 16, 17. In recent years, EMT has been closely associated with expression and regulation of immune checkpoint molecules in several cancers including lung cancer 18-22, breast cancer 23-26, oesophageal cancer 27, gastric cancer 27-29, salivary adenoid cystic carcinoma 30, pancreatic cancer 31, 32 and head and neck cancer 33. Previously, we reported an association between the process of EMT and the expression of several immune checkpoint molecules including PD-L1 in HCC 11, 34. Notably, in HCC patients a close association between EMT and immune checkpoint molecules prognosticated a poor survival 11, 34.

We have previously shown that cytokine, tumour necrosis factor (TNF)-α*,* regulates expression of immune checkpoint molecules in HCC 34. In this study, we have used another cytokine, transforming growth factor (TGF)-β1, which is a potent inducer of EMT in HCC cells 35-37. Our study utilises a reversible EMT model to examine the association between TGF-β1-driven EMT and regulation of immune checkpoint molecules.

## Materials and methods

### Cell culture and reagents

Human HCC cell line PLC/PRF/5 was purchased from CellBank Australia (85061113) and Hep3B cells were sourced from Prof. V. Nathan Subramaniam, The Queensland University of Technology, Australia. Both cell lines were STR profiled and mycoplasma-tested using the MycoAlert test (Abm, Canada) and cultured as previously described 34. The cytokine TGF-β1 was purchased from Peprotech, Australia.

### EMT reversal assay

An *in vitro* reversible EMT model was utilised to evaluate the interaction between immune checkpoint molecule expression and EMT as previously described 34. Briefly, the HCC cells were treated with TGF-β1 for three days and reversal was induced by growing the cells in a TGF-β1 free culture medium for another three days to reverse EMT effects or induce MET. Photographs were taken for cells under all three conditions (Control, EMT and MET) using an inverted microscope equipped with a digital camera (Olympus DP21, Japan).

### Wound healing assay

Cells were seeded into a 24 well plate and allowed to reach confluency. A scratch or wound was made using a sterile pipette tip and washed with 1XPBS. 500 µl fresh culture medium was added into the wells and the photographs were taken at the indicated time points under an inverted microscope equipped with a digital camera (Olympus DP21, Japan). For better contrast, images have been enhanced by false colour background using Fiji plug-in for Image J software version 1.53c.

### Transwell migration assay

Transwell chambers (8 µm pore size, Corning, Australia) were utilised for the transwell migration assay. 1X10^5^ cells were seeded into the upper chamber in a serum free DMEM culture medium. 500 µl of DMEM culture medium with 10% FBS was added into the lower chamber as the chemoattractant. After 24 h incubation at 37 °C, cells were fixed with 4% paraformaldehyde (Fisher Scientific, Australia) for 15 min. The fixed cells were then stained with 0.1% Crystal Violet (Sigma-Aldrich). The cells on the upper chamber were removed and the migrated cells on the lower chamber were photographed under an inverted microscope equipped with a digital camera (Olympus DP21, Japan).

### RNA extraction and cDNA synthesis

RNA extraction was performed with Isolate II Bioline RNA synthesis kit (Bioline, Australia) as per the manufacturer's protocol as described previously 38. Nanodrop 2000c (Thermofisher, Australia) was utilised to confirm the purity and concentration of RNA. Bioline SensiFAST cDNA synthesis kit (Bioline, Australia) was used to reverse transcribe 1 µg RNA into cDNA.

### Quantitative reverse transcription-PCR (qRT-PCR)

qRT-PCR was performed using Lo-ROX SYBR Green (Bioline, Australia) on a ViiA7 Applied Biosystems Real-Time PCR system as described previously 38. Data was analysed using the 2ΔΔCt method where *ActB* was assigned as the housekeeping gene. In this 2ΔΔCt method, target gene expression was normalized to *ActB* expression and data are presented as copies of target gene per 10,000 copies of *ActB*. The sequence for the primers used for qRT-PCR is detailed in previous study 34. The primer sequence not mentioned is listed as follows: *Snai1* forward: GCTGCAGGACTCTAATCCAGA; *Snai1* reverse: ATCTCCGGAGGTGGGATG.

### Protein isolation and western blot analysis

The protein isolation and western blot analysis were performed as previously described 34. Briefly, cells cultured and treated in 6 well plates were lysed using RIPA buffer (Thermofisher, Australia) with Complete (Roche, Australia) and phosSTOP (Roche, Australia) protease and phosphatase inhibitors at 4^o^C to isolate the total protein. A Pierce BCA protein assay kit (Thermofisher, Australia) was used to measure the total protein extracted. 10 µg of protein was used for separation by electrophoresis (SDS-PAGE) in a polyacrylamide gel in the presence of sodium dodecyl sulphate (SDS) and transferred to a polyvinylidene difluoride film (PVDF) membrane. After blocking with 5% skim milk in Tris-buffered saline containing 0.1% Tween 20 (TBS-T), the membranes were incubated with primary antibodies at 4 °C overnight. The protein was detected using an enhanced chemiluminescence reagent, SuperSignal West Femto Maximum Sensitivity Substrate (Thermofisher, Australia) following incubation with HRP-conjugated secondary antibodies. Glyceraldehyde 3-phosphate dehydrogenase (GAPDH) was used as the housekeeping control. Quant LAS 500 was used to capture images and quantified with Image Studio Lite Ver 5.2 software. Antibodies used are listed in previous study 34 and β-Actin (Cat. No. 4967s, Cell Signaling) at dilution of 1:2000.

### Immunofluorescence

Immunofluorescence staining was performed as previously described 34. Briefly, the cells were cultured in 8 well tissue culture treated chamber slides. The cells were then washed with PBS, fixed with 4% paraformaldehyde (Fisher scientific, Australia) for 15 min and permeabilized with 0.1% Triton X-100 (Sigma-Aldrich, Australia). 5% FBS was used as blocking buffer and then the cells were incubated overnight with primary antibodies at 4 °C. The cells were then washed with PBS, incubated with secondary antibodies at room temperature followed by 10 min incubation with 4',6-diamidino-2-phenylindole (DAPI) (Thermofisher Scientific, Australia). The slides were then mounted in ProLong Diamond (Thermofisher Scientific, Australia) for observation with Nikon C2 system and captured and analysed with NIS-Elements software (Nikon, Australia). Antibodies used are listed in previous study 34.

### B7-H3 knockdown

Cells were transfected at 50% confluency for transient siRNA transfection using a control siRNA (4390843) (Thermofisher, Australia), GAPDH siRNA (4390849) and two different silencer select siRNAs targeting B7-H3 (s37290 and s37288) (Thermofisher, Australia) at 10 nM final concentration with Lipofectamine RNAiMAX (Invitrogen, Australia) according to manufacturer's protocol. Cells were incubated with siRNA complex for 72 hours and then collected for further experiments.

### SurvExpress bioinformatics tool

HCC patient datasets were subjected to survival analyses with SurvExpress tool, as previously described 11. Briefly, the HCC patient database, TCGA-Liver-Cancer with 422 patients was analysed 11, 39-41. Using cox regression analyses, we evaluated the coordinate gene expression of TGF-β1 and immune checkpoint molecules to explore their association with the survival of HCC patients 11. The survival times was assessed with Kaplan-Meier curves 11.

### Statistical analysis

Experimental data are presented as mean with standard error of mean (SEM). Prism software version 8.00 (GraphPad Software Inc) was used for the statistical analyses. One-way analysis of variance (ANOVA) followed by Dunnet's multiple comparisons test was used for analysis of dose concentration and time course experiments. Comparisons of were performed using In addition, ANOVA followed by Sidak's multiple comparisons test was used for comparisons of TGF-β1-induced EMT and MET following reversal assay. Gene expression differences between control and TGF-β1 treated cells were analysed using Student's *t*-test. Statistical significance was set at P<0.05. Error bars indicate standard error of the mean (SEM).

A log-rank test was used for testing the P value of survival curves for analysis of survival with SurvExpress. Deviance residuals were applied for the correlation coefficient 11, 42. Cox model was applied to estimate the hazard ratio (HR) between the groups 11.

## Results

### Human HCC cells undergo EMT with TGF-β1 treatment

To examine the induction of EMT by TGF-β1, Hep3B and PLC/PRF/5 cells were treated with various doses of TGF-β1 at different time points. The induction of EMT was evaluated by decreased expression of epithelial markers (*E-cadherin* and *Occludin*) and increased expression of mesenchymal markers (*N-cadherin*, *Vimentin*, *Snai1* and *Fibronectin*). We observed an optimal induction of EMT in Hep3B cells at the concentration of 10 ng/ml of TGF-β1 (Supplementary Fig. S1A) and thus we utilised 10 ng/ml of TGF-β1 for EMT induction in Hep3B cells throughout this study. In addition, we observed robust EMT changes at 72 h post treatment of Hep3B cells with 10 ng/ml of TGF-β1 with the maximum decrease in the expression of epithelial markers *E-cadherin* and *Occludin* and the maximum increase in the expression of majority of mesenchymal markers *N-cadherin*, *Vimentin* and *Snai1* (Supplementary Fig. S1B) and selected the time point of 72 h for TGF-β1 treatment as optimum for this study. Similarly, we also demonstrated that TGF-β1 induces optimal EMT in PLC/PRF/5 cells treated for 72 h with a concentration of 10 ng/ml of TGF-β1 (Supplementary Fig. S2A). In addition, time course experiment reported optimal EMT induction with 10 ng/ml of TGF-β1 treatment in PLC/PRF/5 cells for 72 h (Supplementary Fig. S2B). The induction of EMT in both Hep3B and PLC/PRF/5 cells treated with TGF-β1 was further validated by western blot analysis (Supplementary Fig. S3A and B). Both cell lines demonstrated an upregulation of epithelial markers and downregulation of mesenchymal markers at protein level consistent with EMT changes.

### TGF-β1 induces upregulation of immune checkpoint molecules

**Figure 1:**
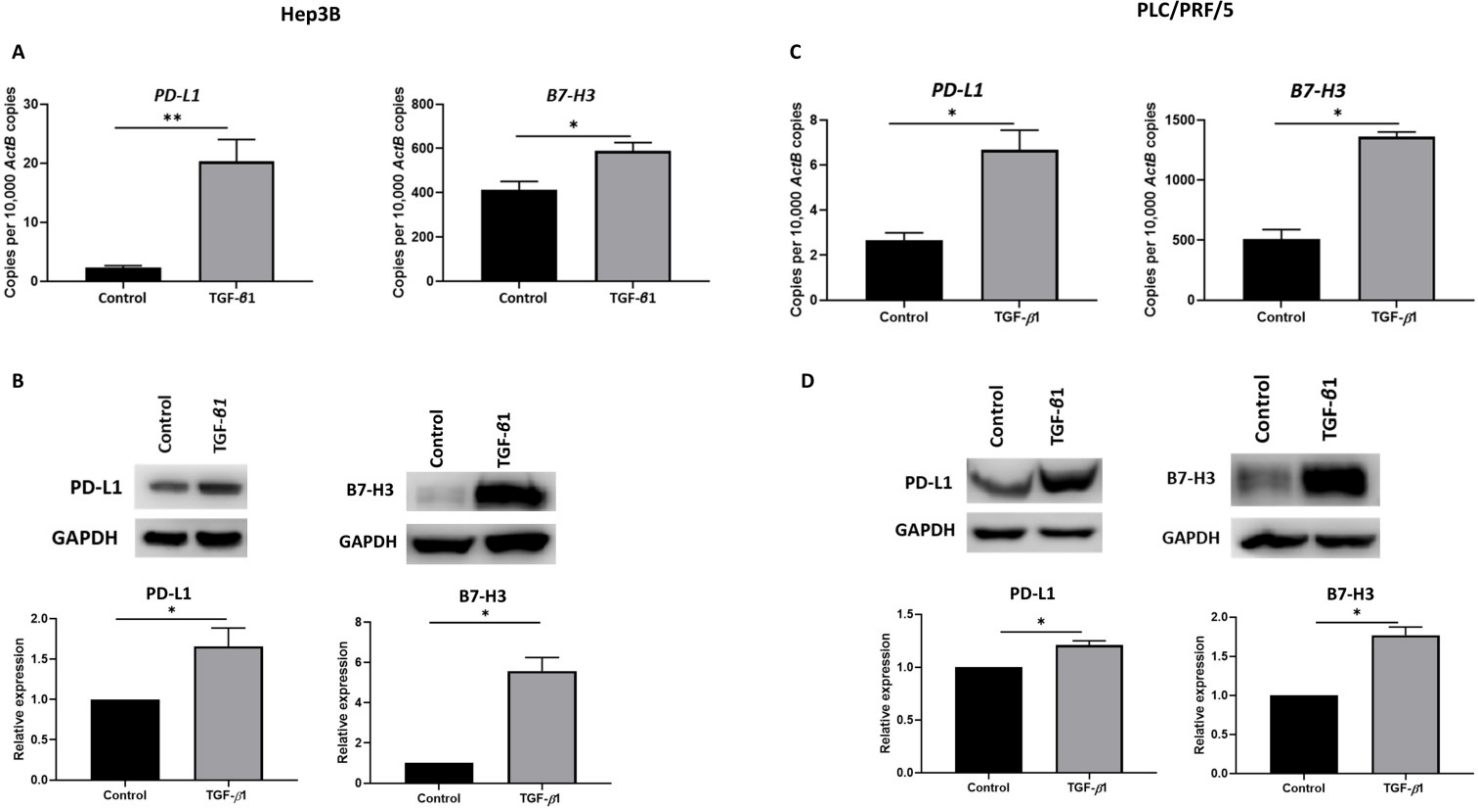
** EMT induced by TGF-*β*1 modulates expression of immune checkpoint molecules in HCC cells.** (A) qRT-PCR revealed upregulation of *PD-L1* and *B7-H3* upon treatment with 10 ng/ml of TGF-*β*1 for 72 h in Hep3B cells. (B) Western blot analysis revealed upregulated expression of PD-L1 and B7-H3 upon treatment with 10 ng/ml of TGF-*β*1 for 72 h in Hep3B cells. (C) qRT-PCR revealed upregulation of *PD-L1* and *B7-H3* upon treatment with 10 ng/ml of TGF-*β*1 for 72 h in PLC/PRF/5 cells. (D) Western blot analysis revealed upregulated expression of PD-L1 and B7-H3 upon treatment with10 ng/ml of TGF-*β*1 for 72 h in PLC/PRF/5 cells*. (n*=3, *p<0.05, **p<0.01). GAPDH was used as loading control.

Previously, we have shown that immune checkpoint modulators, *PD-L1* and *B7-H3* prognosticates a poor outcome in HCC patients 11. To determine the effect of TGF-β1-mediated EMT on expression of these immune checkpoint molecules, we treated Hep3B and PLC/PRF/5 cells with 10 ng/ml of TGF-β1 for 72 h. We observed the upregulation of these immune checkpoint molecules in both Hep3B (Fig. 1A-B) and PLC/PRF/5 (Fig. 1C-D) cells as demonstrated by qRT-PCR and western blot analysis.

### Abrogation of migratory ability of HCC cells upon reversal of TGF-β1-induced EMT

**Figure 2:**
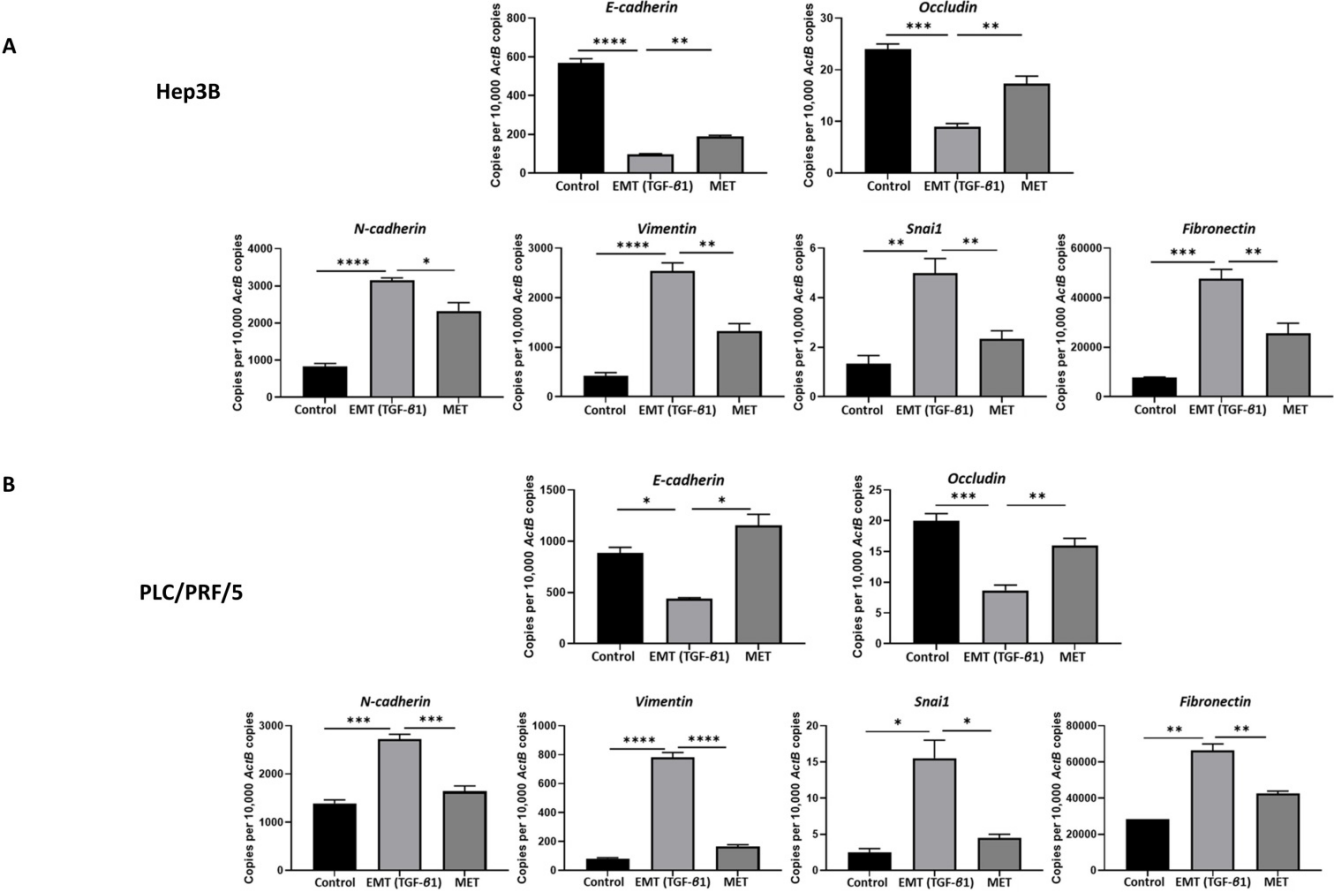
** EMT mediated by TGF-*β*1 in HCC cells is reversible.** qRT-PCR revealed upregulation of *E-cadherin* and *Occludin* and downregulation of *N-cadherin*, *Vimentin*, *Snai1* and *Fibronectin* following EMT reversal assay in (A) Hep3B and (B) PLC/PRF/5 cells (*n*=3, *p<0.05, **p<0.01, ***p<0.005, ****p<0.001).

**Figure 3:**
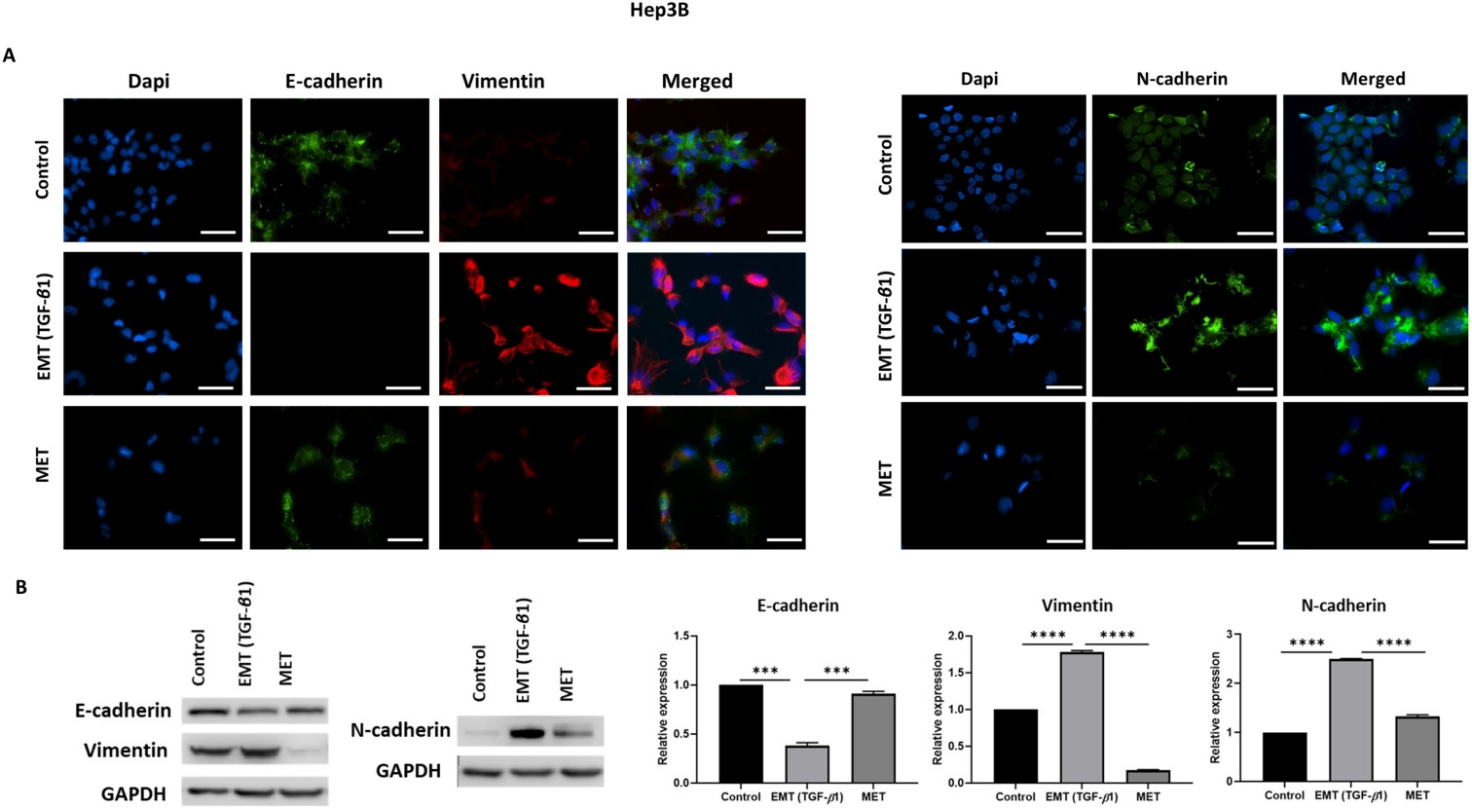
** TGF-*β*1 induces reversible EMT in Hep3B cells.** (A) Fluorescence microscopy demonstrated lower expression of E-cadherin and higher expression of Vimentin and N-cadherin during EMT whereas higher expression of E-cadherin and lower expression of Vimentin and N-cadherin was observed following MET (scale bar = 200 µm, magnification 20X). (B) Western blot analysis revealed decreased expression of E-cadherin and increased expression of Vimentin and N-cadherin during EMT and upregulation of E-cadherin and downregulation of Vimentin and N-cadherin during reversal or MET (*n*=3, ***p<0.005, ****p<0.001). GAPDH was used as loading control.

**Figure 4:**
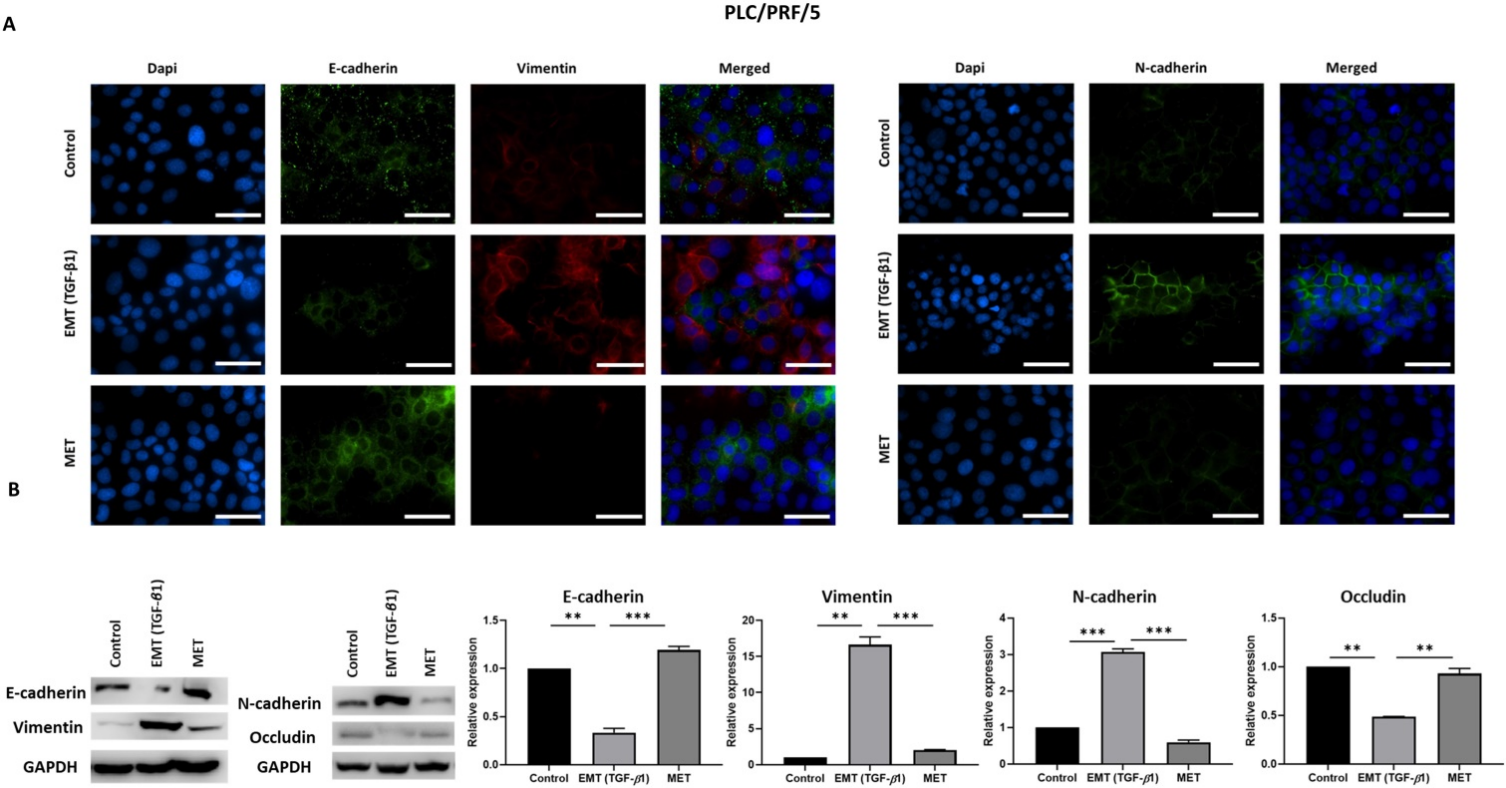
** TGF-*β*1-mediated EMT is reversible in PLC/PRF/5 cells.** (A) Fluorescence microscopy revealed decreased expression of E-cadherin and increased expression of Vimentin and N-cadherin during EMT whereas increased expression of E-cadherin and decreased expression of Vimentin and N-cadherin was observed following MET (scale bar = 200 µm, magnification 20X). (B) Western blot analysis revealed downregulation of E-cadherin and Occludin and upregulation of Vimentin and N-cadherin during EMT and upregulation of E-cadherin and downregulation of Vimentin and N-cadherin during MET. (*n*=3, **p<0.01, ***p<0.005). GAPDH was used as loading control.

We utilised an *in vitro* EMT reversal model to induce EMT in a reversible manner in HCC cells. The qRT-PCR results demonstrated the occurrence of EMT post TGF-β1 treatment consistent with the reduced expression of epithelial markers (*E-cadherin* and *Occludin*) and concomitant increased expression of mesenchymal markers (*N-cadherin*, *Vimentin*, *Snai1* and *Fibronectin*). The removal of TGF-β1 induced the cells to undergo an MET consistent with upregulation of epithelial markers (*E-cadherin and Occludin*) and downregulation of mesenchymal markers (*N-cadherin, Vimentin, Snai1 and Fibronectin*) in Hep3B (Fig. 2A) and PLC/PRF/5 (Fig. 2B) cells. The reversible process of EMT and MET was further confirmed by immunofluorescence and western blot analysis in Hep3B (Fig. 3A-B) and PLC/PRF/5 (Fig. 4A-B) cells.

**Figure 5:**
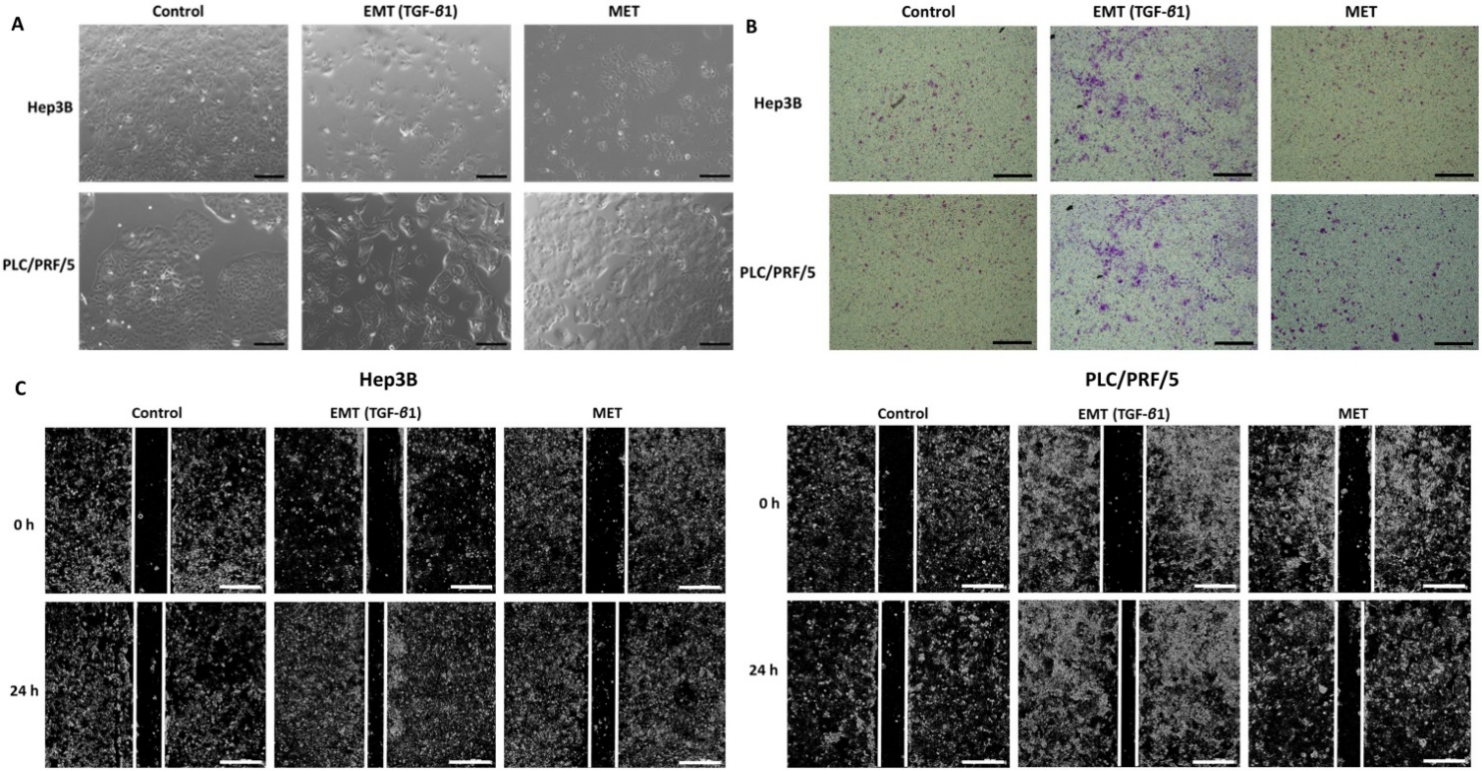
** TGF-*β*1-mediated EMT modulates migratory ability of HCC cells.** (A) Morphology changes in Hep3B and PLC/PRF/5 cells during EMT and MET (scale bar = 500 µm, magnification 10X). (B) Transwell migration assay revealed increased motility of Hep3B and PLC/PRF/5 cells upon TGF-*β*1-induced EMT and decreased motility following MET (scale bar = 500 µm, magnification 10X). (C) The wound healing assay demonstrated that the migratory ability is increased upon EMT and decreased following MET in Hep3B and PLC/PRF/5 cells (scale bar = 500 µm, magnification 10X).

As the process of EMT is involved in tumour metastasis, the migratory ability of HCC cells were compared upon treatment with TGF-β1 and reversal assay using wound healing and transwell migration assay along with observation of morphology changes. We observed morphological changes in both Hep3B and PLC/PRF/5 during the process of EMT and MET (Fig. 5A). During EMT, cells appear to be fibroblast-like and elongated and during MET, they display a cobblestone morphology similar to the untreated control cells. Furthermore, we observed that TGF-*β*1-induced EMT enhances the migratory ability of Hep3B and PLC/PRF/5 cells whereas the reversal assay reduces the motility both Hep3B and PLC/PRF/5 cells as demonstrated by transwell migration assay (Fig. 5B) and wound healing assay (Fig. 5C). The EMT marker changes together with the functional changes observed in HCC cells in the reversible EMT model makes this assay a robust *in vitro* tool to investigate the changes associated with the EMT status.

### Reversal of EMT reverses expression of immune checkpoint molecules

**Figure 6:**
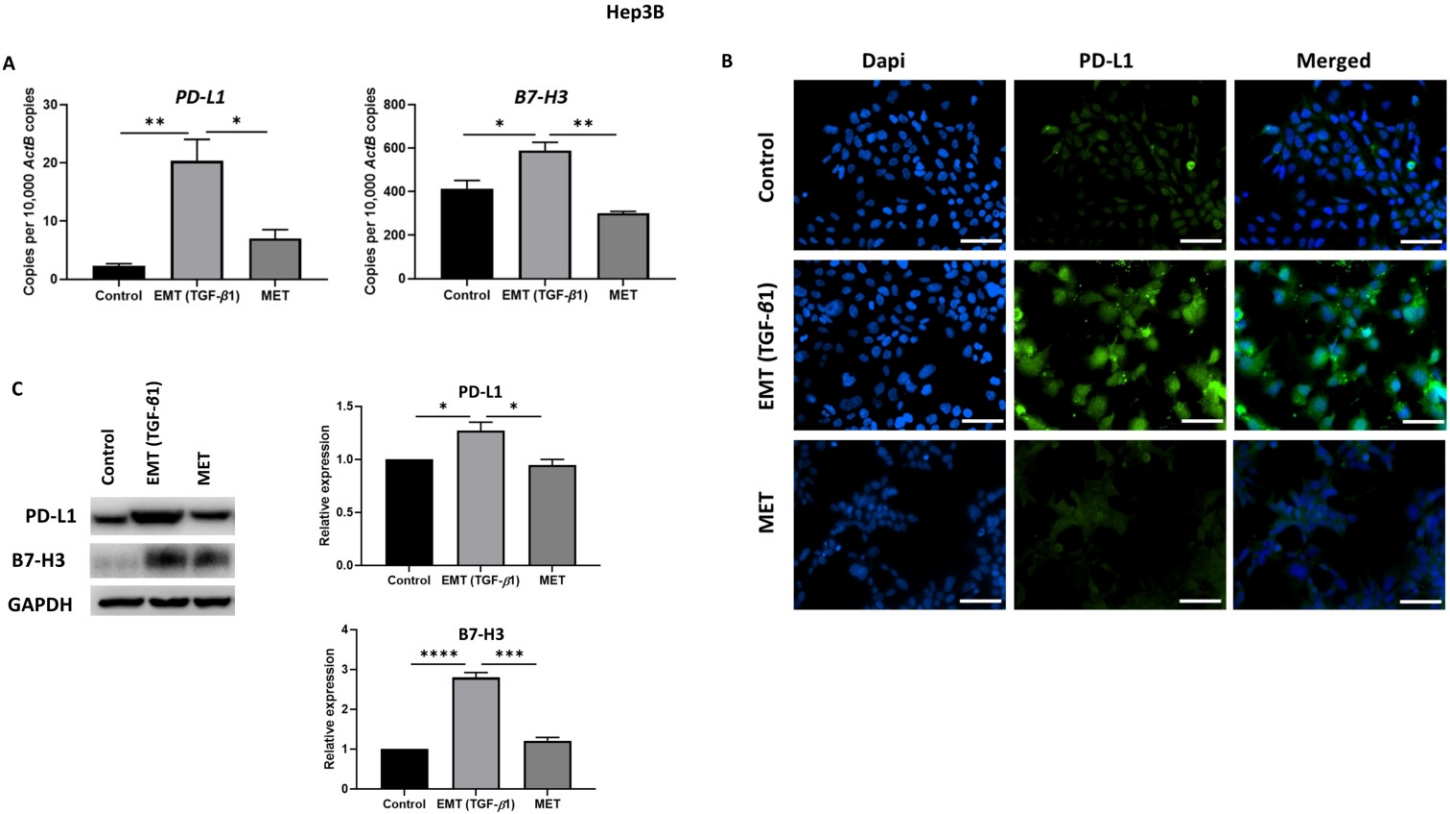
** Expression of immune checkpoint molecules is reversed following reversal of EMT in Hep3B cells.** (A) qRT-PCR, (B) fluorescence microscopy (scale bar = 200 µm, magnification 20X) and (C) western blot analysis demonstrated upregulation of PD-L1 and B7-H3 upon induction of EMT by TGF-*β*1 and downregulation of PD-L1 and B7-H3 upon reversal of MET. (*n*=3, * p<0.05, **p<0.01, ***p<0.005, ****p<0.001). GAPDH was used as loading control.

**Figure 7:**
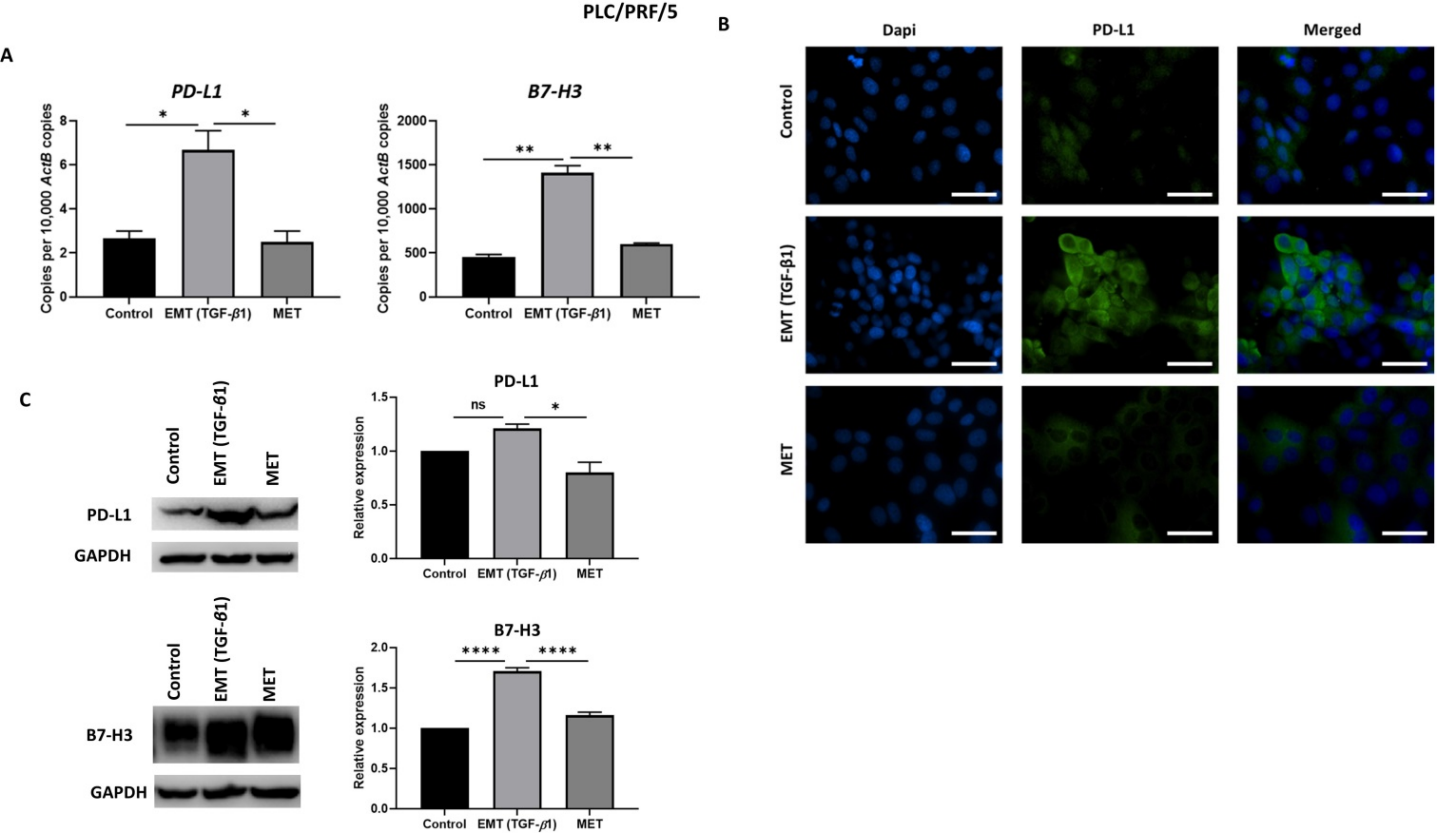
** Expression of immune checkpoint molecules is reversed following reversal of EMT in PLC/PRF/5 cells.** (A) qRT-PCR, (B) fluorescence microscopy (scale bar = 200 µm, magnification 20X) and (C) western blot analysis demonstrated upregulation of PD-L1 and B7-H3 upon induction of EMT by TGF-*β*1 and downregulation of PD-L1 and B7-H3 upon reversal of MET. (*n*=3, *p<0.05, **p<0.01, ****p<0.001). GAPDH was used as loading control.

To examine whether the expression of immune checkpoint molecules were altered with EMT status, we utilised the reversible EMT assay. We observed downregulation of PD-L1 and B7-H3 following EMT reversal assay as demonstrated by qRT-PCR, immunofluorescence staining and western blot analysis in Hep3B (Fig. 6A-C) and PLC/PRF/5 (Fig. 7A-C) cells. The changes in EMT status affects the expression of immune checkpoint molecules suggesting a role for TGF-β1 in the regulation of immune checkpoint molecules PD-L1 and B7-H3.

### Knockdown of B7-H3 can reverse TGF-β1-induced EMT in HCC cells

**Figure 8:**
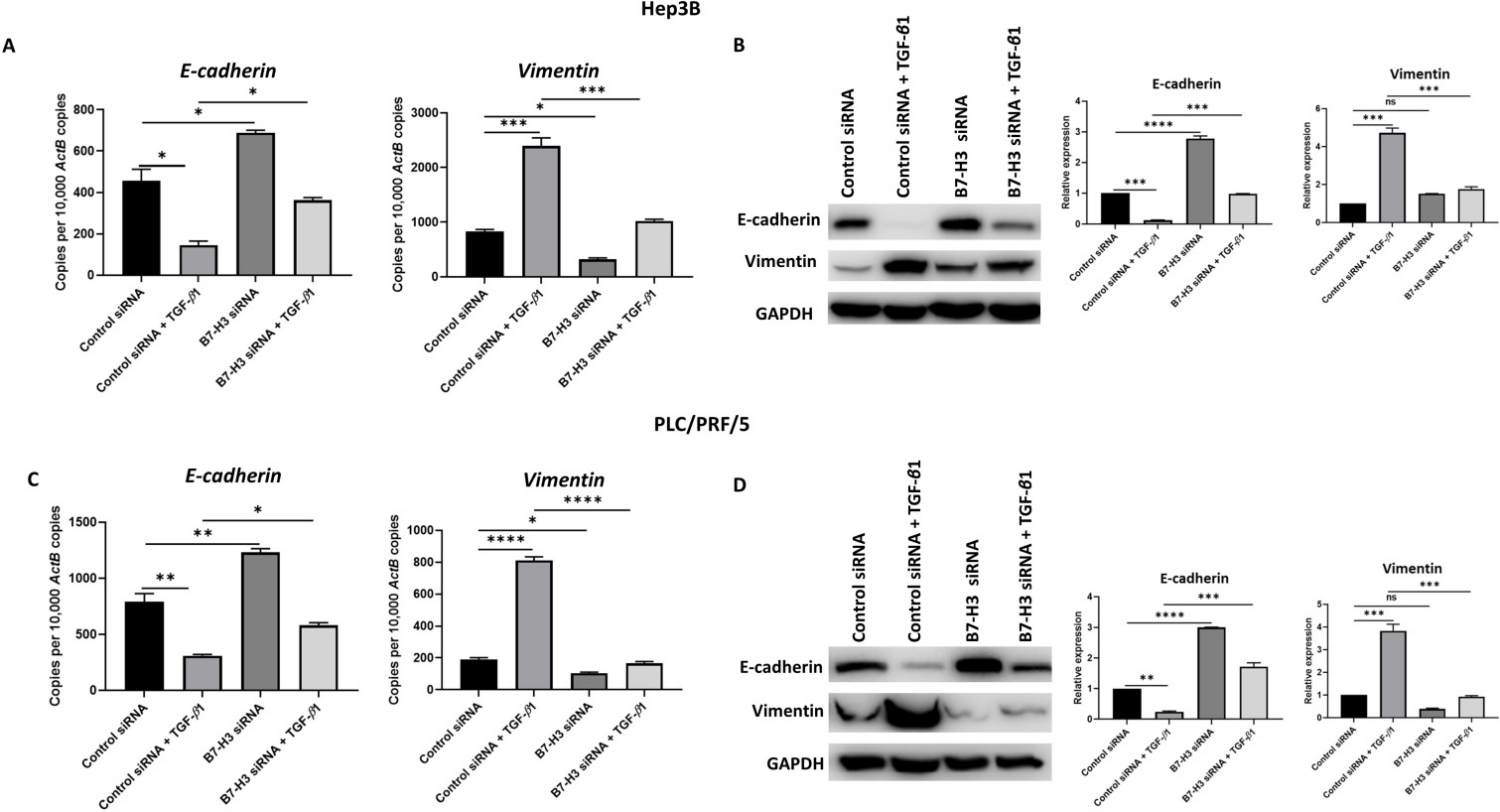
** Knockdown of B7-H3 can reverse TGF-*β*1-induced EMT in HCC cells.** siRNA-mediated silencing of B7-H3 expression in Hep3B cells resulted in reversal of TGF-β1-induced EMT as demonstrated by (A) qRT-PCR and (B) western blot analysis. Knockdown of B7-H3 expression in PLC/PRF/5 cells caused reversal of TGF-β1-induced EMT as demonstrated by (C) qRT-PCR and (D) western blot analysis. GAPDH was used as loading control. (*n*=3, *p<0.05, **p<0.01, ***p<0.005, ****p<0.001).

To further validate the association between TGF-β1-induced EMT and expression of immune checkpoint molecules such as B7-H3, we utilised two different siRNAs to silence the expression of B7-H3. The efficacy of B7-H3 knockdown was confirmed by qRT-PCR and western blot analysis in both Hep3B (Supplementary Fig. S4A-B) and PLC/PRF/5 (Supplementary Fig. S4C-D) cells. Next, we examined the effects of siRNA-mediated silencing of B7-H3 on expression of EMT markers to validate the aforementioned association between EMT and immune checkpoint expression. Interestingly, we observed reversal of TGF-β1-mediated EMT following B7-H3 knockdown evident by upregulation of epithelial marker E-cadherin and downregulation of mesenchymal marker Vimentin as revealed by qRT-PCR and western blot analysis in both Hep3B (Fig. 8A-B) and PLC/PRF/5 (Fig. 8C-D) cells.

**Figure 9:**
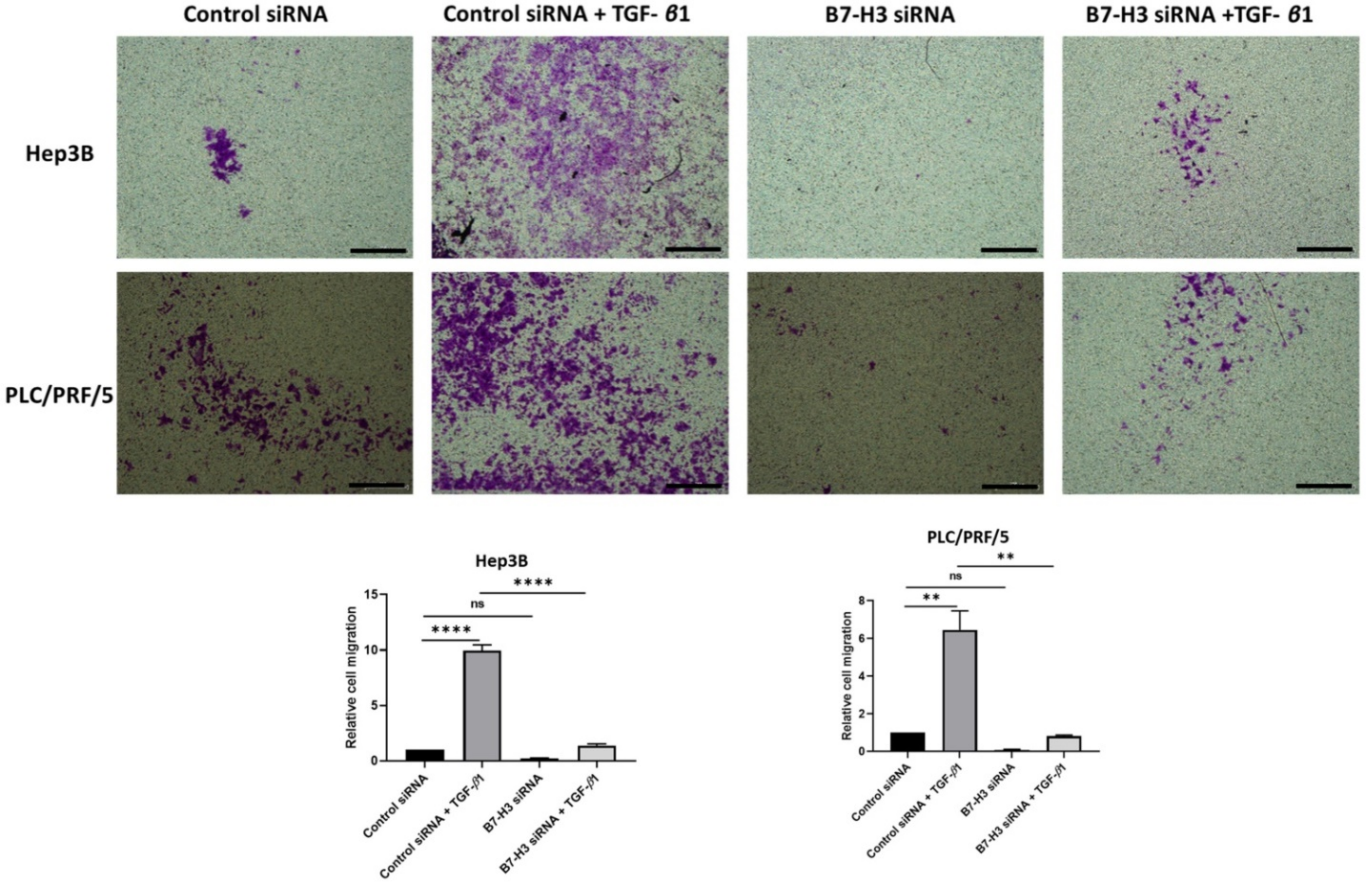
** Silencing of B7-H3 reduces motility of HCC cells.** Transwell migration revealed that number of cells migrating reduced significantly following B7-H3 knockdown in both Hep3B and PLC/PRF/5 cells (scale bar = 500 µm, magnification 10X) (*n*=3, **p<0.01, ****p<0.001).

This relationship between TGF-β1-mediated EMT and expression of immune checkpoint molecules was further validated at functional level with transwell migration. We observed reduced migration of cells following siRNA-mediated knockdown of B7-H3 (Fig. 9). This further justified our hypothesis on the significant association between TGF-β1-mediated EMT and immune checkpoint molecule expression in HCC.

### Coordinate expression of TGF-β1 and immune checkpoint molecules in HCC patients

**Figure 10:**
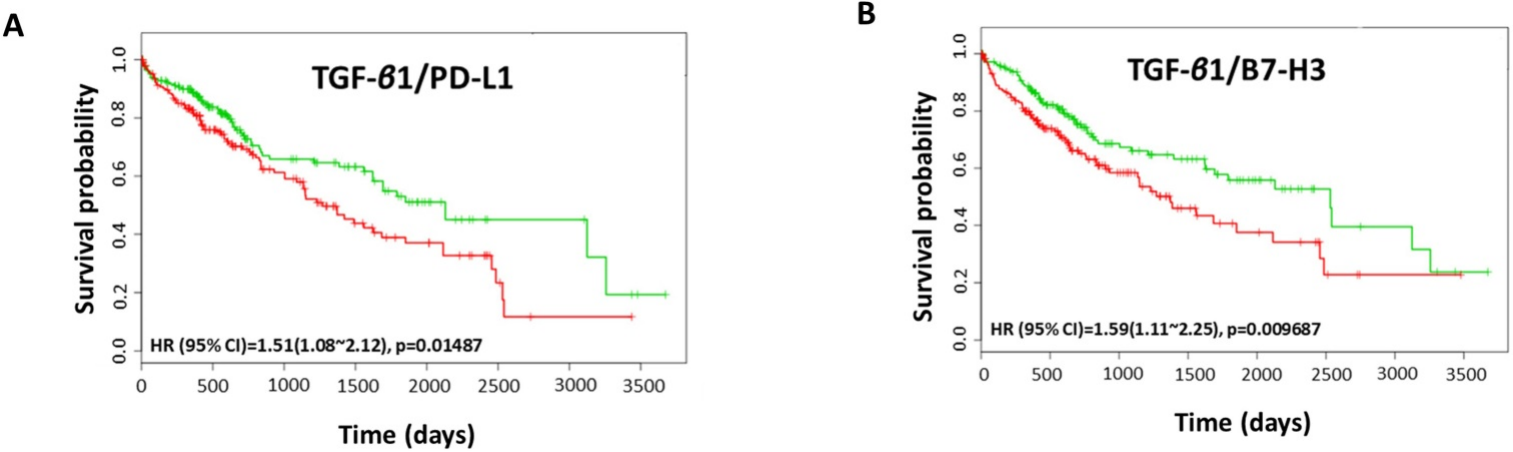
** Coordinate expression of TGF-*β*1 and immune checkpoint molecules in HCC patients.** Kaplan-Meier survival curves demonstrating the (A) gene expression of TGF-*β*1/PD-L1 and overall survival, and (B) gene expression of TGF-*β*1/B7-H3 and overall survival in HCC patient samples. Red curve represents high-risk group, while green curve represents low-risk group 11. Markers (+) represent censoring samples. X-axis represents the study time in days. Y-axis represents survival probability.

We utilised TCGA-Liver-Cancer HCC database with 422 HCC patients within Survexpress to assess the association between coordinate expression of TGF-β1 and immune checkpoint molecules with overall survival in HCC patients. We observed that the coordinate expression of TGF-β1 in combination with PD-L1 resulted in a significantly poorer overall survival in 422 patients (HR: 1.51, CI: 1.08~2.12, Log-Rank Equal Curves p=0.01487) (Fig. 10A). Similarly, the combination of TGF-β1 with B7-H3 showed significant poorer overall survival (HR: 1.59, CI: 1.11~2.25, Log-Rank Equal Curves p=0.009687) (Fig. 10B). This data suggests that a significant correlation of TGF-β1 with PD-L1 and B7-H3 in HCC patients prognosticates a poor outcome.

## Discussion

In this study, we explored the relationship between TGF-β1-induced EMT and expression of immune checkpoint molecules in human HCC cells based on a reversible model of EMT. We observed induction of EMT and upregulation of PD-L1 and B7-H3 in both Hep3B and PLC/PRF/5 cells along with enhanced motility upon treatment with TGF-β1. The close association of EMT and immune checkpoints in HCC was further confirmed by reversibility of the expression of immune checkpoint molecules during EMT and MET. In addition, we also observed that HCC patients with coordinate expression of TGF-β1 and immune checkpoint molecules PD-L1 or B7-H3 had poor overall survival.

TGF-β signalling plays a key role in the process of EMT and carcinogenesis in several cancers 43, 44. In hepatocarcinogenesis, TGF-β signalling has been implicated in all the stages of disease progression, from inflammation to cirrhosis to carcinoma 45, 46. TGF-β is a widely studied EMT inducer that regulates EMT-transcription factors (EMT-TFs) 46, 47. Several *in vitro* studies have utilised TGF-β for inducing EMT in HCC 37, 48-50. In addition, studies have demonstrated reversible induction of EMT with cytokine TNF-α 34 or combination of TGF-β1 and TNF-α 18. The present study utilised TGF-β1 as a reversible EMT inducer in human HCC cells, Hep3B and PLC/PRF/5. Our study is the first to explore the association of TGF-β1-induced EMT and expression of immune checkpoint molecules in HCC.

Immunotherapeutic approaches based on ICIs have revolutionized the cancer treatment scenario by modulating the patient's immune system to combat the tumour 51, 52. Several ICI drugs targeting CTLA-4 or PD-1/PD-L1 interaction have been approved by the FDA for various cancer types including melanoma, lung cancer, renal cell carcinoma, breast cancer, head and neck cancer, colorectal cancer, urothelial cancer and HCC 18-33. Despite the approval of few ICIs and several ongoing clinical trials, ICIs as a monotherapy have failed to show promising result as first-line treatment in HCC compared to Sorafenib 53, 54. In addition, the response of HCC patients to ICIs may be affected by several molecular features including genetic alterations, variation in inflammatory infiltrates, microsatellite instability (MSI) and expression of alternative immune checkpoint molecules 53, 55, 56. Thus, there is a need for identification of robust predictive biomarkers along with combined ICI therapeutic approaches for enhanced clinical outcome in HCC patients 53.

Tissue-agnostic biomarkers such as tumour mutation burden (TMB) and MSI have been examined for cancer immunotherapy 57-59. TMB has been utilised as a predictive biomarker in cancer immunotherapy in several studies 11, 60-62. Our previous study demonstrated significant association of TMB with poor clinical outcomes in HCC patients 11. Recently, FDA has approved Pembrolizumab, an anti-PD-1 ICI, for the treatment of tumour mutational burden-high (TMB-H) cancers based on KEYNOTE-158 study 63. This approval of Pembrolizumab for TMB-H cancers is subsequent to previous FDA approval of the drug in MSI-high (MSI-H) or mismatch repair deficient (dMMR) cancers 64. Thus, genetic and immunogenic features of tumour should be considered with use of tissue-agnostic biomarkers before the selection of ICIs for cancer immunotherapy.

The HCC TME includes cancer cells, immune cells [T-cells, B-cells, dendritic cells, myeloid-derived suppressor cells (MDSCs), tumor-associated macrophages (TAMs)], carcinoma-associated fibroblasts, endothelial cells, hepatic stellate cells, and the extracellular matrix (ECM) 65. As the TME is a crucial facilitator of HCC progression and resistance to immunotherapies, much of the recent research is focused on the various components of the TME to identify predictive and prognostic biomarkers for ICI treatment in HCC patients 56, 66. For instance, a study developed a prognostic biomarker for immunotherapy comprising of eight gene risk signature based on hypoxia status in the TME of HCC patient 56. Another study reported CD38 expression within the TME including tumour and certain immune subsets, such as macrophages as a predictive marker of responsiveness to anti-PD-1/PD-L1 single agent treatment in HCC patients 66. HCC TME is rich source of soluble factors and cytokines that can regulate the expression of immune checkpoints and can be utilised as prognostic biomarkers 67. Thus, further investigation of potential biomarkers that predict the efficacy of ICI treatment in HCC patients is of the utmost importance. Furthermore, it is important to examine the mechanism underlying the poor response and therapy resistance to ICI monotherapies to improve therapeutic efficacy in cancer therapy.The process of EMT is known to influence immune evasion of tumour cells contributing to immunosuppression through regulation of immune checkpoint molecules 52, 68, 69. EMT is closely associated with immune checkpoint-dependent immunosuppression resulting in aggressive and drug resistant tumours 52, 70-72. As previously mentioned, EMT is known to regulate immune checkpoint molecules, in particular PD-L1, in several cancers. In recent years, studies have explored several aspects of EMT process responsible for regulation of immune checkpoint molecules, tumour microenvironment and prognosis 33, 70, 73, 74.

This study reported that TGF-β1-mediated EMT promotes migratory ability and enhances expression of PD-L1 and B7-H3 in human HCC cells, Hep3B and PLC/PRF/5. In HCC, we have previously reported a significant association of PD-L1 expression with EMT phenotype characterised by lower expression of epithelial marker E-cadherin and higher expression of mesenchymal marker Vimentin in 422 patients 11. Other studies have reported a close association between EMT and PD-L1 in various cancers. A study by Asgarova *et al* reported that cytokines TNF-α and TGF-β1 regulates PD-L1 expression in non-small cell lung carcinoma 18. A study in gastric carcinoma demonstrated that TGF-β1 promotes motility and PD-L1 expression via NF-κB activation 28. Similarly, another study revealed that TGF-β1-mediated EMT enhances PD-L1 expression in head and neck squamous cell carcinoma 33. Furthermore, a study in pancreatic carcinoma revealed that TGF-β upregulates the expression of PD-L1 via both Smad 2/3-dependent and independent pathways along with increased migration and invasion *in vitro* 32. The study also reported that PD-L1 expression induces Snail transcription factors which results in TGF-β-mediated EMT 32. Likewise, another interesting study by David *et al* examined PD-L1 expression through TGF-β-induced EMT by utilizing M7824, a bifunctional fusion protein, inhibiting TGF-β and PD-L1 19. Besides EMT associated with TGF-β, the regulation of immune checkpoints are associated with some inflammatory cytokines such as interferon-γ (IFN-γ), TNF-*α* and interleukin-17 (IL-17) in various cancer types 31, 75. Studies have reported other mechanisms of EMT that regulates PD-L1 expression such as EGF-induced EGFR activation 30; ZEB1 and miR-200-mediated EMT 26, 70, Ataxia Telangiectasia Mutated (ATM) through JAK/STAT3 signalling activation 22.

In addition to PD-L1, we have reported TGF-β1-induced EMT mediated upregulation of another immune checkpoint molecule, B7-H3 in HCC cells 34. We have previously reported upregulation of B7-H3 expression via TNF-α-mediated EMT in HCC cells 34. B7-H3 is often overexpressed in cancer types characterised by increased proliferation and invasive potential such as liver, bladder, esophageal, breast, cervical, glioma, colorectal and gastric cancer 76. A study in HCC revealed that B7-H3 promotes metastasis and invasion of HCC cells by undergoing EMT via JAK2/STAT2/Slug signalling pathway and elevated B7-H3 expression is correlated with poor clinical outcomes in HCC patients 77. B7-H3 has also been reported to induce EMT and cancer stemness in colorectal cancer 78, induce cell invasion and sphere formation in glioma cells 79 and regulate cancer-initiating cells in ovarian cancer 80.

EMT is known to be significantly associated with overexpression of multiple immune checkpoint molecules resulting in immunosuppression 21. A study demonstrated close link between EMT score and expression of panel of immune checkpoint molecules including PD-1, PD-L1, B7-H3, PD-L2 and others 69, 81. A study by Lou et al reported strong association between EMT and upregulation of multiple immune checkpoint molecules including PD-1, CTLA-4, PD-L1, B7-H3, PD-L2, BTLA, and TIM-3 in lung adenocarcinoma 82. Another study in head and neck squamous cell carcinoma also suggested that an EMT gene signature with a total of 82 genes was closely associated with prognosis and expression of immune checkpoint molecules 33. Similarly Thompson *et al* suggested that gene signatures reflecting immune infiltration and EMT may be crucial in predicting patient's response to immunotherapy in lung cancer 83.

A few other studies have explored the regulation of immune checkpoint molecules in HCC by cytokines. These studies did not evaluate the EMT status and the role of EMT in modulating immune checkpoint molecules. One of the studies demonstrated that combined blocking of TGF-β and PD-L1 improved the immune response against tumour 84. It has been reported that TNF-α and IFN-γ enhance the expression of PD-L1 synergistically in HCC cells 85.

Failure of immune checkpoint therapy also results from expression of alternative immune checkpoint molecules such as TIM-3, LAG-3, IDO-1 and VISTA 55. Brown et al have reported resistance to ICIs (anti-PD-1 and anti-CTLA-4) through upregulation of alternative immune checkpoint IDO-1 in HCC patients 86. Inhibition of IDO-1 resulted in enhancing the therapeutic efficacy of ICIs suggesting combination approach in HCC 86.

Our study provides valuable insight into regulation of immune checkpoint molecules PD-L1 and B7-H3 through TGF-β1-induced EMT that may result in an aggressive tumour phenotype and therapy resistance. Furthermore, HCC patients showing coordinate expression of TGF-*β*1 with PD-L1 or B7-H3 have poor overall survival. Hence, it is conceivable that by using combination approach by targeting TGF-β1-induced EMT along with ICIs such as PD-L1 and/or B7-H3 may lead to better clinical outcome in HCC patients. Moreover, this study suggests that EMT markers may be useful biomarkers to predict patient response in ICI immunotherapy as EMT status is closely associated with the expression of immune checkpoint molecules. However, further studies to determine molecular mechanisms involved in regulation of immune checkpoints and its association with EMT are warranted.

## Supplementary Material

Supplementary figures.

## Abbreviations

HCC: hepatocellular carcinoma
EMT: epithelial-to-mesenchymal transition
TGF: transforming growth factor
PD-L1: programmed cell death protein ligand-1
FDA: food and drug administration
ICI: immune checkpoint inhibitor
PD-1: programmed cell death protein-1
SDS: sodium dodecyl sulphate
PVDF: polyvinylidene difluoride film
GAPDH: glyceraldehyde 3-phosphate dehydrogenase
DAPI: 4',6-diamidino-2-phenylindole
SEM: standard error of mean
TMB: tumour mutation burden
TME: tumour microenvironment
MSI: microsatellite instability
TMB-H: tumour mutation burden high
MSI-H: microsatellite instability high
dMMR: mismatch repair deficient

## References

[1] Singal AG, Lampertico P, Nahon P (2020). Epidemiology and surveillance for hepatocellular carcinoma: New trends. J Hepatol.

[2] Villanueva A (2019). Hepatocellular Carcinoma. N Engl J Med.

[3] Akinyemiju T, Abera S, Ahmed M, Alam N, Alemayohu MA, Allen C (2017). The Burden of Primary Liver Cancer and Underlying Etiologies From 1990 to 2015 at the Global, Regional, and National Level: Results From the Global Burden of Disease Study 2015. JAMA Oncol.

[4] Dal Bo M, De Mattia E, Baboci L, Mezzalira S, Cecchin E, Assaraf YG (2020). New insights into the pharmacological, immunological, and CAR-T-cell approaches in the treatment of hepatocellular carcinoma. Drug Resist Updat.

[5] Brahmer J, Reckamp KL, Baas P, Crinò L, Eberhardt WE, Poddubskaya E (2015). Nivolumab versus Docetaxel in Advanced Squamous-Cell Non-Small-Cell Lung Cancer. N Engl J Med.

[6] Hato T, Goyal L, Greten TF, Duda DG, Zhu AX (2014). Immune checkpoint blockade in hepatocellular carcinoma: current progress and future directions. Hepatology.

[7] Weber JS, D'Angelo SP, Minor D, Hodi FS, Gutzmer R, Neyns B (2015). Nivolumab versus chemotherapy in patients with advanced melanoma who progressed after anti-CTLA-4 treatment (CheckMate 037): a randomised, controlled, open-label, phase 3 trial. Lancet Oncol.

[8] Yau T, Kang YK, Kim TY, El-Khoueiry AB, Santoro A, Sangro B (2020). Efficacy and Safety of Nivolumab Plus Ipilimumab in Patients With Advanced Hepatocellular Carcinoma Previously Treated With Sorafenib: The CheckMate 040 Randomized Clinical Trial. JAMA Oncol.

[9] Cheng AL, Qin S, Ikeda M, Galle P, Ducreux M, Zhu A (2019). IMbrave150: Efficacy and safety results from a ph III study evaluating atezolizumab (atezo) 1 bevacizumab (bev) vs sorafenib (Sor) as first treatment (tx) for patients (pts) with unresectable hepatocellular carcinoma (HCC). Annals of Oncology.

[10] Li Z, Li N, Li F, Zhou Z, Sang J, Chen Y (2016). Immune checkpoint proteins PD-1 and TIM-3 are both highly expressed in liver tissues and correlate with their gene polymorphisms in patients with HBV-related hepatocellular carcinoma. Medicine (Baltimore).

[11] Shrestha R, Prithviraj P, Anaka M, Bridle KR, Crawford DHG, Dhungel B (2018). Monitoring Immune Checkpoint Regulators as Predictive Biomarkers in Hepatocellular Carcinoma. Front Oncol.

[12] Jayachandran A, Dhungel B, Steel JC (2016). Epithelial-to-mesenchymal plasticity of cancer stem cells: therapeutic targets in hepatocellular carcinoma. J Hematol Oncol.

[13] van Zijl F, Zulehner G, Petz M, Schneller D, Kornauth C, Hau M (2009). Epithelial-mesenchymal transition in hepatocellular carcinoma. Future Oncol.

[14] Yan L, Xu F, Dai CL (2018). Relationship between epithelial-to-mesenchymal transition and the inflammatory microenvironment of hepatocellular carcinoma. J Exp Clin Cancer Res.

[15] Mir N, Jayachandran A, Dhungel B, Shrestha R, Steel JC (2017). Epithelial-to-Mesenchymal Transition: A Mediator of Sorafenib Resistance in Advanced Hepatocellular Carcinoma. Curr Cancer Drug Targets.

[16] Chaffer CL, Thompson EW, Williams ED (2007). Mesenchymal to epithelial transition in development and disease. Cells Tissues Organs.

[17] Hugo H, Ackland ML, Blick T, Lawrence MG, Clements JA, Williams ED (2007). Epithelial-mesenchymal and mesenchymal-epithelial transitions in carcinoma progression. J Cell Physiol.

[18] Asgarova A, Asgarov K, Godet Y, Peixoto P, Nadaradjane A, Boyer-Guittaut M (2018). PD-L1 expression is regulated by both DNA methylation and NF-kB during EMT signaling in non-small cell lung carcinoma. Oncoimmunology.

[19] David JM, Dominguez C, Palena C (2017). Pharmacological and immunological targeting of tumor mesenchymalization. Pharmacol Ther.

[20] Li F, Zhu T, Yue Y, Zhu X, Wang J, Liang L (2018). Preliminary mechanisms of regulating PD-L1 expression in non-small cell lung cancer during the EMT process. Oncol Rep.

[21] Manjunath Y, Upparahalli SV, Avella DM, Deroche CB, Kimchi ET, Staveley-O'Carroll KF (2019). PD-L1 Expression with Epithelial Mesenchymal Transition of Circulating Tumor Cells Is Associated with Poor Survival in Curatively Resected Non-Small Cell Lung Cancer. Cancers (Basel).

[22] Shen M, Xu Z, Xu W, Jiang K, Zhang F, Ding Q (2019). Inhibition of ATM reverses EMT and decreases metastatic potential of cisplatin-resistant lung cancer cells through JAK/STAT3/PD-L1 pathway. J Exp Clin Cancer Res.

[23] Alsuliman A, Colak D, Al-Harazi O, Fitwi H, Tulbah A, Al-Tweigeri T (2015). Bidirectional crosstalk between PD-L1 expression and epithelial to mesenchymal transition: significance in claudin-low breast cancer cells. Mol Cancer.

[24] Dongre A, Rashidian M, Reinhardt F, Bagnato A, Keckesova Z, Ploegh HL (2017). Epithelial-to-Mesenchymal Transition Contributes to Immunosuppression in Breast Carcinomas. Cancer Res.

[25] Kumar S, Davra V, Obr AE, Geng K, Wood TL, De Lorenzo MS (2017). Crk adaptor protein promotes PD-L1 expression, EMT and immune evasion in a murine model of triple-negative breast cancer. Oncoimmunology.

[26] Noman MZ, Janji B, Abdou A, Hasmim M, Terry S, Tan TZ (2017). The immune checkpoint ligand PD-L1 is upregulated in EMT-activated human breast cancer cells by a mechanism involving ZEB-1 and miR-200. Oncoimmunology.

[27] Chen L, Xiong Y, Li J, Zheng X, Zhou Q, Turner A (2017). PD-L1 Expression Promotes Epithelial to Mesenchymal Transition in Human Esophageal Cancer. Cell Physiol Biochem.

[28] Xu D, Li J, Li RY, Lan T, Xiao C, Gong P (2019). PD-L1 Expression Is Regulated By NF-κB During EMT Signaling In Gastric Carcinoma. Onco Targets Ther.

[29] Xu Z, Gu C, Yao X, Guo W, Wang H, Lin T (2020). CD73 promotes tumor metastasis by modulating RICS/RhoA signaling and EMT in gastric cancer. Cell Death Dis.

[30] Wang Y, Hu J, Ye W, Zhang X, Ju H, Xu D (2018). EGFR activation induced Snail-dependent EMT and myc-dependent PD-L1 in human salivary adenoid cystic carcinoma cells. Cell Cycle.

[31] Imai D, Yoshizumi T, Okano S, Itoh S, Ikegami T, Harada N (2019). IFN-γ Promotes Epithelial-Mesenchymal Transition and the Expression of PD-L1 in Pancreatic Cancer. J Surg Res.

[32] Kang JH, Jung MY, Leof EB (2019). B7-1 drives TGF-β stimulated pancreatic carcinoma cell migration and expression of EMT target genes. PLoS One.

[33] Jung AR, Jung CH, Noh JK, Lee YC, Eun YG (2020). Epithelial-mesenchymal transition gene signature is associated with prognosis and tumor microenvironment in head and neck squamous cell carcinoma. Sci Rep.

[34] Shrestha R, Bridle KR, Crawford DHG, Jayachandran A (2020). TNF-α-mediated epithelial-to-mesenchymal transition regulates expression of immune checkpoint molecules in hepatocellular carcinoma. Mol Med Rep.

[35] Liu L, Li N, Zhang Q, Zhou J, Lin L, He X (2017). Inhibition of ERK1/2 Signaling Impairs the Promoting Effects of TGF-β1 on Hepatocellular Carcinoma Cell Invasion and Epithelial-Mesenchymal Transition. Oncol Res.

[36] Rawal P, Siddiqui H, Hassan M, Choudhary MC, Tripathi DM, Nain V (2019). Endothelial Cell-Derived TGF-β Promotes Epithelial-Mesenchymal Transition via CD133 in HBx-Infected Hepatoma Cells. Front Oncol.

[37] Wang B, Liu T, Wu JC, Luo SZ, Chen R, Lu LG (2018). STAT3 aggravates TGF-β1-induced hepatic epithelial-to-mesenchymal transition and migration. Biomed Pharmacother.

[38] Jayachandran A, Shrestha R, Dhungel B, Huang IT, Vasconcelos MYK, Morrison BJ (2017). Murine hepatocellular carcinoma derived stem cells reveal epithelial-to-mesenchymal plasticity. World J Stem Cells.

[39] Hoshida Y, Nijman SM, Kobayashi M, Chan JA, Brunet JP, Chiang DY (2009). Integrative transcriptome analysis reveals common molecular subclasses of human hepatocellular carcinoma. Cancer Res.

[40] Hoshida Y, Villanueva A, Kobayashi M, Peix J, Chiang DY, Camargo A (2008). Gene expression in fixed tissues and outcome in hepatocellular carcinoma. N Engl J Med.

[41] Tsuchiya M, Parker JS, Kono H, Matsuda M, Fujii H, Rusyn I (2010). Gene expression in nontumoral liver tissue and recurrence-free survival in hepatitis C virus-positive hepatocellular carcinoma. Mol Cancer.

[42] Awan FM, Naz A, Obaid A, Ali A, Ahmad J, Anjum S (2015). Identification of Circulating Biomarker Candidates for Hepatocellular Carcinoma (HCC): An Integrated Prioritization Approach. PLoS One.

[43] Thiery JP, Acloque H, Huang RY, Nieto MA (2009). Epithelial-mesenchymal transitions in development and disease. Cell.

[44] Xu J, Lamouille S, Derynck R (2009). TGF-beta-induced epithelial to mesenchymal transition. Cell Res.

[45] Dooley S, ten Dijke P (2012). TGF-β in progression of liver disease. Cell Tissue Res.

[46] Fabregat I, Caballero-Díaz D (2018). Transforming Growth Factor-β-Induced Cell Plasticity in Liver Fibrosis and Hepatocarcinogenesis. Front Oncol.

[47] Nieto MA, Huang RY, Jackson RA, Thiery JP (2016). EMT: 2016. Cell.

[48] Huang J, Qiu M, Wan L, Wang G, Huang T, Chen Z (2018). TGF-β1 Promotes Hepatocellular Carcinoma Invasion and Metastasis via ERK Pathway-Mediated FGFR4 Expression. Cell Physiol Biochem.

[49] Malfettone A, Soukupova J, Bertran E, Crosas-Molist E, Lastra R, Fernando J (2017). Transforming growth factor-β-induced plasticity causes a migratory stemness phenotype in hepatocellular carcinoma. Cancer Lett.

[50] Xu Z, Shen MX, Ma DZ, Wang LY, Zha XL (2003). TGF-beta1-promoted epithelial-to-mesenchymal transformation and cell adhesion contribute to TGF-beta1-enhanced cell migration in SMMC-7721 cells. Cell Res.

[51] Gentles AJ, Newman AM, Liu CL, Bratman SV, Feng W, Kim D (2015). The prognostic landscape of genes and infiltrating immune cells across human cancers. Nat Med.

[52] Soundararajan R, Fradette JJ, Konen JM, Moulder S, Zhang X, Gibbons DL (2019). Targeting the Interplay between Epithelial-to-Mesenchymal-Transition and the Immune System for Effective Immunotherapy. Cancers (Basel).

[53] Nishida N, Kudo M (2020). Immune Phenotype and Immune Checkpoint Inhibitors for the Treatment of Human Hepatocellular Carcinoma. Cancers (Basel).

[54] Yau T, Park JW, Finn RS, Cheng AL, Mathurin P, Edeline J (2019). CheckMate 459: A randomized, multi-center phase III study of nivolumab (NIVO) vs sorafenib (SOR) as first-line (1L) treatment in patients (pts) with advanced hepatocellular carcinoma (aHCC). Annals of Oncology.

[55] Xu F, Jin T, Zhu Y, Dai C (2018). Immune checkpoint therapy in liver cancer. J Exp Clin Cancer Res.

[56] Liu Z, Liu L, Lu T, Wang L, Li Z, Jiao D (2021). Hypoxia Molecular Characterization in Hepatocellular Carcinoma Identifies One Risk Signature and Two Nomograms for Clinical Management. J Oncol.

[57] Ang C, Klempner SJ, Ali SM, Madison R, Ross JS, Severson EA (2019). Prevalence of established and emerging biomarkers of immune checkpoint inhibitor response in advanced hepatocellular carcinoma. Oncotarget.

[58] Kim SI, Cassella CR, Byrne KT (2020). Tumor Burden and Immunotherapy: Impact on Immune Infiltration and Therapeutic Outcomes. Front Immunol.

[59] Sung WWY, Chow JCH, Cho WCS (2020). Tumor mutational burden as a tissue-agnostic biomarker for cancer immunotherapy. Expert Rev Clin Pharmacol.

[60] Kim H, Hong JY, Lee J, Park SH, Park JO, Park YS (2021). Clinical sequencing to assess tumor mutational burden as a useful biomarker to immunotherapy in various solid tumors. Ther Adv Med Oncol.

[61] Wong CN, Fessas P, Dominy K, Mauri FA, Kaneko T, Parcq PD (2021). Qualification of tumour mutational burden by targeted next-generation sequencing as a biomarker in hepatocellular carcinoma. Liver Int.

[62] Yin L, Zhou L, Xu R (2020). Identification of Tumor Mutation Burden and Immune Infiltrates in Hepatocellular Carcinoma Based on Multi-Omics Analysis. Front Mol Biosci.

[63] Marabelle A, Fakih M, Lopez J, Shah M, Shapira-Frommer R, Nakagawa K (2020). Association of tumour mutational burden with outcomes in patients with advanced solid tumours treated with pembrolizumab: prospective biomarker analysis of the multicohort, open-label, phase 2 KEYNOTE-158 study. Lancet Oncol.

[64] Prasad V, Kaestner V, Mailankody S (2018). Cancer Drugs Approved Based on Biomarkers and Not Tumor Type-FDA Approval of Pembrolizumab for Mismatch Repair-Deficient Solid Cancers. JAMA Oncol.

[65] Zhang FP, Huang YP, Luo WX, Deng WY, Liu CQ, Xu LB (2020). Construction of a risk score prognosis model based on hepatocellular carcinoma microenvironment. World J Gastroenterol.

[66] Ng HHM, Lee RY, Goh S, Tay ISY, Lim X, Lee B (2020). Immunohistochemical scoring of CD38 in the tumor microenvironment predicts responsiveness to anti-PD-1/PD-L1 immunotherapy in hepatocellular carcinoma. J Immunother Cancer.

[67] Hilmi M, Neuzillet C, Calderaro J, Lafdil F, Pawlotsky JM, Rousseau B (2019). Angiogenesis and immune checkpoint inhibitors as therapies for hepatocellular carcinoma: current knowledge and future research directions. J Immunother Cancer.

[68] Kudo-Saito C, Shirako H, Takeuchi T, Kawakami Y (2009). Cancer metastasis is accelerated through immunosuppression during Snail-induced EMT of cancer cells. Cancer Cell.

[69] Shrestha R, Bridle KR, Crawford DHG, Jayachandran A (2019). Immune checkpoint blockade therapies for HCC: current status and future implications. Hepatoma Research. 2019.

[70] Chen L, Gibbons DL, Goswami S, Cortez MA, Ahn YH, Byers LA (2014). Metastasis is regulated via microRNA-200/ZEB1 axis control of tumour cell PD-L1 expression and intratumoral immunosuppression. Nat Commun.

[71] Chouaib S, Janji B, Tittarelli A, Eggermont A, Thiery JP (2014). Tumor plasticity interferes with anti-tumor immunity. Crit Rev Immunol.

[72] Dong P, Xiong Y, Yue J, Hanley SJB, Watari H (2018). Tumor-Intrinsic PD-L1 Signaling in Cancer Initiation, Development and Treatment: Beyond Immune Evasion. Front Oncol.

[73] De Matteis S, Canale M, Verlicchi A, Bronte G, Delmonte A, Crinò L (2019). Advances in Molecular Mechanisms and Immunotherapy Involving the Immune Cell-Promoted Epithelial-to-Mesenchymal Transition in Lung Cancer. J Oncol.

[74] Lequeux A, Noman MZ, Xiao M, Sauvage D, Van Moer K, Viry E (2019). Impact of hypoxic tumor microenvironment and tumor cell plasticity on the expression of immune checkpoints. Cancer Lett.

[75] Wang X, Yang L, Huang F, Zhang Q, Liu S, Ma L (2017). Inflammatory cytokines IL-17 and TNF-α up-regulate PD-L1 expression in human prostate and colon cancer cells. Immunol Lett.

[76] Dong P, Xiong Y, Yue J, Hanley SJB, Watari H (2018). B7H3 As a Promoter of Metastasis and Promising Therapeutic Target. Front Oncol.

[77] Kang FB, Wang L, Jia HC, Li D, Li HJ, Zhang YG (2015). B7-H3 promotes aggression and invasion of hepatocellular carcinoma by targeting epithelial-to-mesenchymal transition via JAK2/STAT3/Slug signaling pathway. Cancer Cell Int.

[78] Jiang B, Zhang T, Liu F, Sun Z, Shi H, Hua D (2016). The co-stimulatory molecule B7-H3 promotes the epithelial-mesenchymal transition in colorectal cancer. Oncotarget.

[79] Lemke D, Pfenning PN, Sahm F, Klein AC, Kempf T, Warnken U (2012). Costimulatory protein 4IgB7H3 drives the malignant phenotype of glioblastoma by mediating immune escape and invasiveness. Clin Cancer Res.

[80] Fauci JM, Sabbatino F, Wang Y, Londoño-Joshi AI, Straughn JM, Landen CN (2014). Monoclonal antibody-based immunotherapy of ovarian cancer: targeting ovarian cancer cells with the B7-H3-specific mAb 376.96. Gynecol Oncol.

[81] Mak MP, Tong P, Diao L, Cardnell RJ, Gibbons DL, William WN (2016). A Patient-Derived, Pan-Cancer EMT Signature Identifies Global Molecular Alterations and Immune Target Enrichment Following Epithelial-to-Mesenchymal Transition. Clin Cancer Res.

[82] Lou Y, Diao L, Cuentas ER, Denning WL, Chen L, Fan YH (2016). Epithelial-Mesenchymal Transition Is Associated with a Distinct Tumor Microenvironment Including Elevation of Inflammatory Signals and Multiple Immune Checkpoints in Lung Adenocarcinoma. Clin Cancer Res.

[83] Thompson JC, Hwang WT, Davis C, Deshpande C, Jeffries S, Rajpurohit Y (2020). Gene signatures of tumor inflammation and epithelial-to-mesenchymal transition (EMT) predict responses to immune checkpoint blockade in lung cancer with high accuracy. Lung Cancer.

[84] Ihling C, Naughton B, Zhang Y, Rolfe PA, Frick-Krieger E, Terracciano LM (2019). Observational Study of PD-L1, TGF-β, and Immune Cell Infiltrates in Hepatocellular Carcinoma. Front Med (Lausanne).

[85] Li N, Wang J, Zhang N, Zhuang M, Zong Z, Zou J (2018). Cross-talk between TNF-α and IFN-γ signaling in induction of B7-H1 expression in hepatocellular carcinoma cells. Cancer Immunol Immunother.

[86] Brown ZJ, Yu SJ, Heinrich B, Ma C, Fu Q, Sandhu M (2018). Indoleamine 2,3-dioxygenase provides adaptive resistance to immune checkpoint inhibitors in hepatocellular carcinoma. Cancer Immunol Immunother.

