# Supplementary material for: Immune checkpoint molecules are regulated by transforming growth factor (TGF)-β1-induced epithelial-to-mesenchymal transition in hepatocellular carcinoma
Source: Int J Med Sci. 2021 Apr 22;18(12):2466–79. doi: 10.7150/ijms.54239 (PMC8176170; doi:10.7150/ijms.54239)
Supplement: Supplementary file 1 — Supplementary figures. [file ijmsv18p2466s1.pdf]

Supplementary Material:

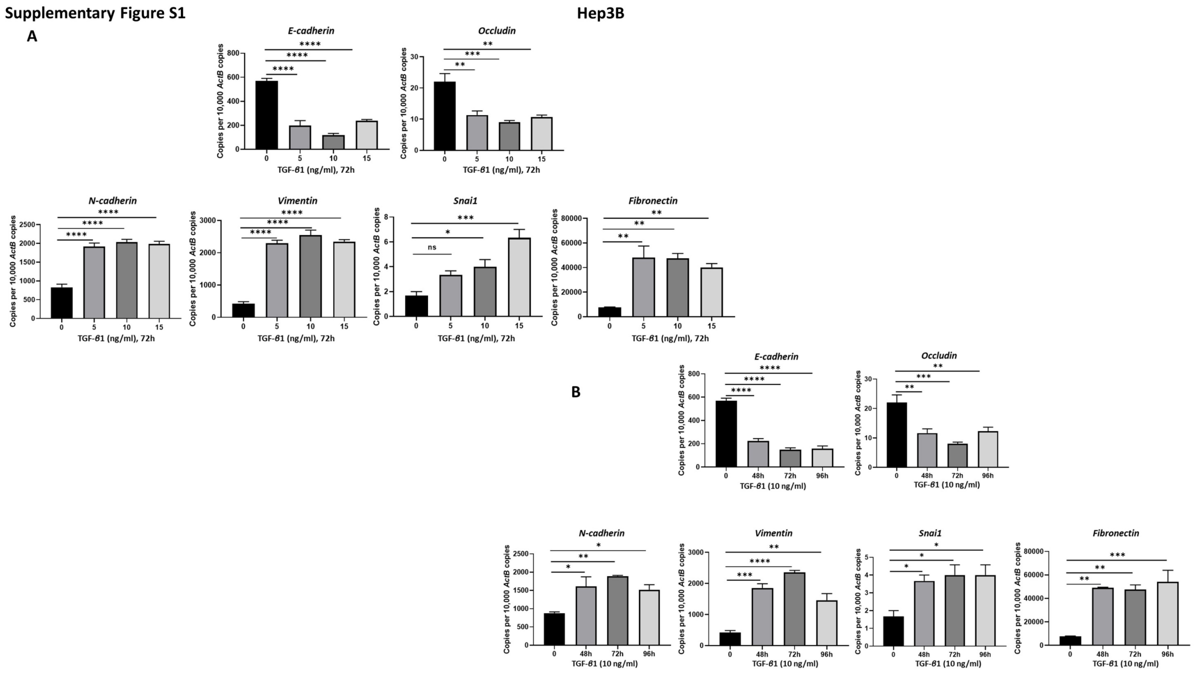

Supplementary Figure S1. TGF-β1 induces EMT in Hep3B cells. qRT-PCR revealed downregulation of *E-cadherin* and *Occludin* and upregulation of *N-cadherin*, *Vimentin*, *Snai1* and *Fibronectin* upon treatment with (A) various doses of TGF-β1 0, 5, 10, 15 ng/ml for 72 h and (B) 10 ng/ml of TGF-β1 at various time points 0, 48 h, 72 h, 96 h (n=3, * $p<0.05$, ** $p<0.01$, *** $p<0.005$, ****$p<0.001$).

Supplementary Figure S2 PLC/PRF/5

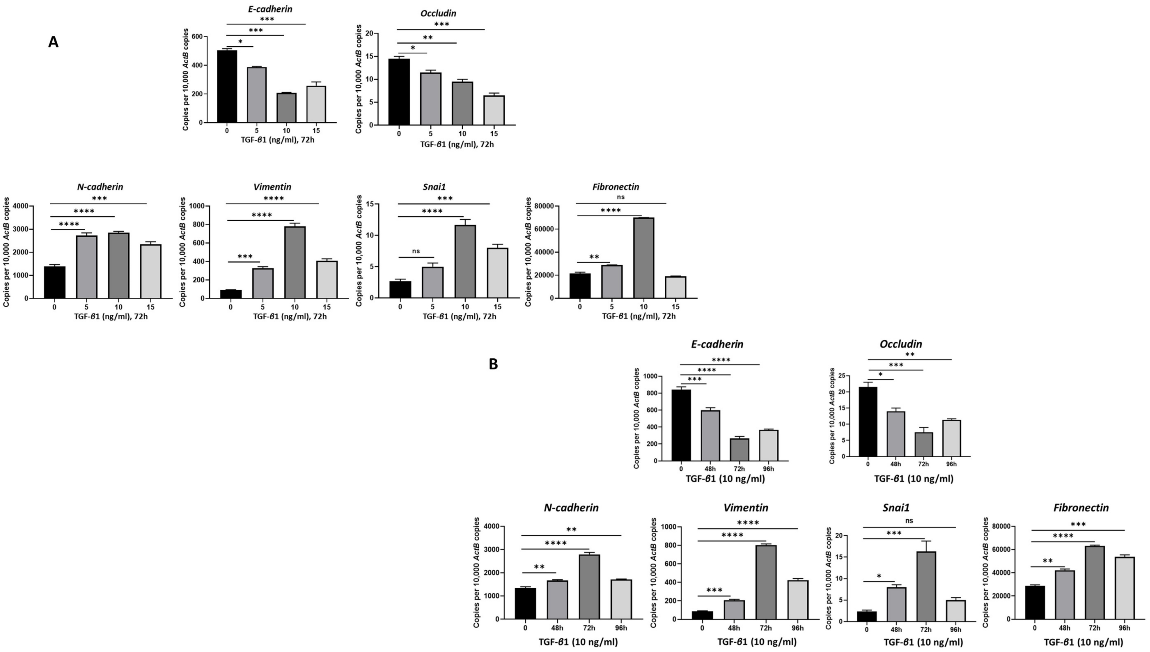

Supplementary Figure S2. TGF-β1 induces EMT in PLC/PRF/5 cells. qRT-PCR demonstrated reduced expression of *E-cadherin* and *Occludin* and elevated expression of *N-cadherin*, *Vimentin*, *Snai1* and *Fibronectin* upon treatment with (A) various doses of TGF-β1 0, 5, 10, 15 ng/ml for 72 h and (B) 10 ng/ml of TGF-β1 at various time points 0, 48 h, 72 h, 96 h (n=3, * p<0.05, ** p<0.01, *** p<0.005, ****p<0.001).

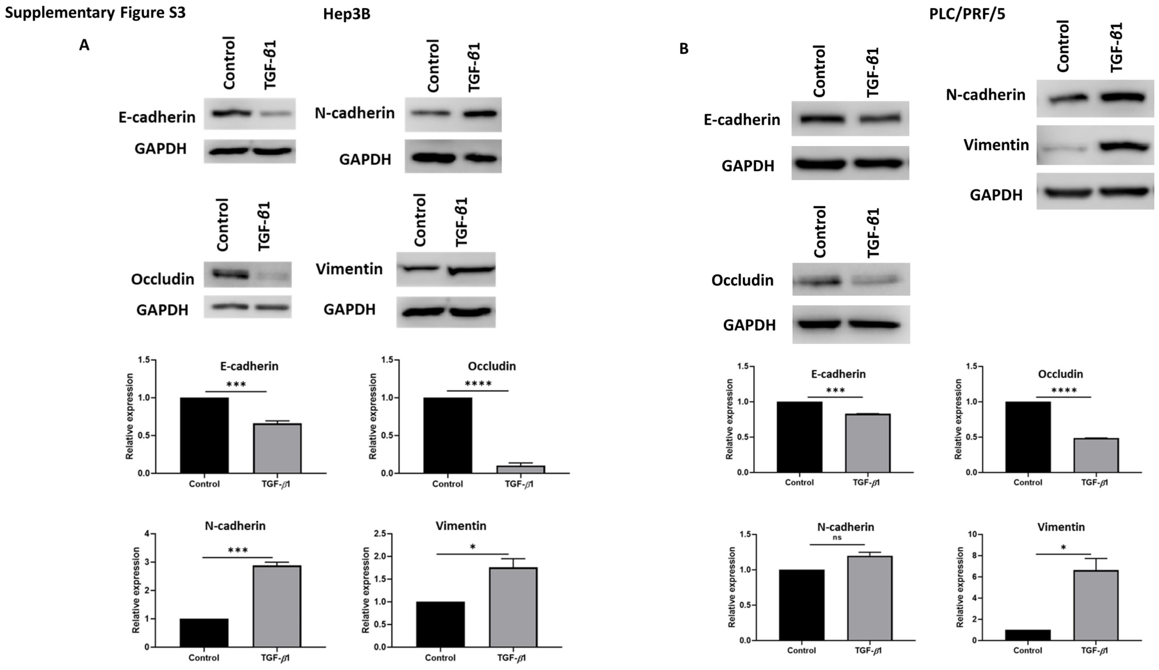

Supplementary Figure S3. TGF-*β*1 induces EMT in human HCC cells. Western blot analysis revealed donwregulation of E-cadherin and Occludin and upregulation of N-cadherin and Vimentin upon treatment with 10 ng/ml of TGF-β1 for 72 h in (A) Hep3B and (B) PLC/PRF/5 cells (n=3, * p<0.05, *** p<0.005, ****p<0.001). GAPDH was used as loading control.

Supplementary Figure S4

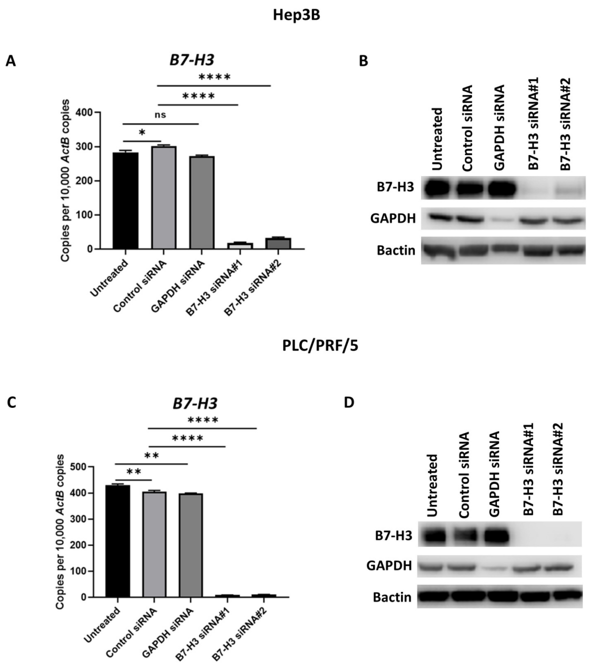

Supplementary Figure S4. Knockdown of B7-H3 in HCC cells. Silencing of B7-H3 with two specific B7-H3 siRNAs in Hep3B cells as demonstrated by (A) qRT-PCR and (B) western blot analysis. Bactin was used as loading control. Knockdown of B7-H3 expression in PLC/PRF/5 cells with two specific siRNAs against B7-H3 as demonstrated by (C) qRT-PCR and (D) western blot analysis. Bactin was used as loading control. GAPDH siRNA transfection was used as positive control for transfection in both cell lines (n=3, * $p<0.05$, ** $p<0.01$, **** $p<0.001$).
